# Melatonin Affects Mitochondrial Fission/Fusion Dynamics in the Diabetic Retina

**DOI:** 10.1155/2019/8463125

**Published:** 2019-04-11

**Authors:** Janet Ya-An Chang, Fei Yu, Liheng Shi, Michael L. Ko, Gladys Y.-P. Ko

**Affiliations:** ^1^Department of Veterinary Integrative Biosciences, Texas A&M University, College Station, Texas, USA; ^2^Interdisciplinary Toxicology Program, Texas A&M University, College Station, Texas, USA; ^3^Texas A&M Institute of Neuroscience, Texas A&M University, College Station, Texas, USA

## Abstract

Mitochondrial fission and fusion are dependent on cellular nutritional states, and maintaining this dynamics is critical for the health of cells. Starvation triggers mitochondrial fusion to maintain bioenergetic efficiency, but during nutrient overloads (as with hyperglycemic conditions), fragmenting mitochondria is a way to store nutrients to avoid waste of energy. In addition to ATP production, mitochondria play an important role in buffering intracellular calcium (Ca^2+^). We found that in cultured 661W cells, a photoreceptor-derived cell line, hyperglycemic conditions triggered an increase of the expression of dynamin-related protein 1 (DRP1), a protein marker of mitochondrial fission, and a decrease of mitofusin 2 (MFN2), a protein for mitochondrial fusion. Further, these hyperglycemic cells also had decreased mitochondrial Ca^2+^ but increased cytosolic Ca^2+^. Treating these hyperglycemic cells with melatonin, a multifaceted antioxidant, averted hyperglycemia-altered mitochondrial fission-and-fusion dynamics and mitochondrial Ca^2+^ levels. To mimic how people most commonly take melatonin supplements, we gave melatonin to streptozotocin- (STZ-) induced type 1 diabetic mice by daily oral gavage and determined the effects of melatonin on diabetic eyes. We found that melatonin was not able to reverse the STZ-induced systemic hyperglycemic condition, but it prevented STZ-induced damage to the neural retina and retinal microvasculature. The beneficial effects of melatonin in the neural retina in part were through alleviating STZ-caused changes in mitochondrial dynamics and Ca^2+^ buffering.

## 1. Introduction

Mitochondria are dynamic organelles that constantly divide and fuse, and maintaining a proper equilibrium in this dynamics is critical in healthy cells [1, 2]. Under starvation, mitochondria can fuse with each other to maintain bioenergetic efficiency [3]. When there is a nutrient overload, fragmenting mitochondria is a way to store nutrients to avoid energy waste [4, 5]. Hence, the number and shape of mitochondria within a cell are tightly associated with cellular metabolism [6]. Mitochondrial fission requires recruiting dynamin-related protein 1 (DRP1) from the cytosol to the outer mitochondrial surface, whereas mitofusin 2 (MFN2) on the outer mitochondrial membrane coordinates with the protein optic atrophy 1 (OPA1) on the inner membrane to regulate mitochondrial fusion [7, 8]. Most mitochondria in the retina are located in the photoreceptors, and mitochondria are a major intracellular source of reactive oxygen species (ROS), a by-product of the mitochondrial respiratory chain [9]. As photoreceptors have the highest metabolic rate and consume more oxygen, they generate more ROS than other retinal cells [10].

In addition to ATP production, mitochondria play an important role to buffer intracellular calcium (Ca^2+^). When the cytosolic Ca^2+^ concentration is elevated due to stimulation, mitochondria along with the endoplasmic reticulum (ER) take up and store Ca^2+^ [11]. Mitochondria also prevent Ca^2+^ depletion in the ER by extruding Ca^2+^ to the cytoplasm [12, 13]. In retinal photoreceptors, mitochondria act as mediators to regulate Ca^2+^ uptake in the outer segment and cell body [14]. The mitochondrial calcium uniporter (MCU) is a highly selective Ca^2+^ channel located in the inner membrane of mitochondria, which allows for the passage of Ca^2+^ into the matrix, and it is primarily responsible for mitochondrial storage of intracellular Ca^2+^ [15–17].

Oxidative stress is a major culprit in the pathogenesis of many metabolic diseases including diabetic retinopathy (DR) [18]. Thus, controlling the source of oxidative stress is critical in DR management. Diabetic retinopathy is a dual disorder with microvascular complications and retinal degeneration [19]. As for the role of mitochondria and ROS in DR, retinal endothelial cells isolated from type 2 diabetic patients have increased mitochondrial fission and ROS overproduction [20], and the retina from DR patients also shows downregulated mitochondrial fusion [21]. Historically, DR has been investigated and treated as a complication of the vasculature [22, 23]. However, increasing evidence shows that retinal neural dysfunction precedes any microvascular complication [24]. In animal models, pharmacological or genetic induction of photoreceptor death in early diabetes dampens the generation of ROS and stops the progression of DR [25], suggesting that photoreceptors are the major source of intraocular oxidative stress under diabetic insults and contribute to the vascular lesions and pathogenesis of early DR [9, 26]. Previously, we found that in the streptozotocin- (STZ-) induced diabetic retina, calcium homeostasis is impaired, and signaling pathways that are involved in calcium homeostasis are downregulated [27]. However, it is not known whether the impaired calcium homeostasis under diabetic stress is in part due to the damage to mitochondrial Ca^2+^ buffering. Furthermore, whether the balance of mitochondrial fission/fusion dynamics is impaired in photoreceptors under hyperglycemic stress is not clear.

Melatonin is a strong antioxidant that can scavenge a variety of ROS, including hydroxyl radical, H_2_O_2_, O^2−^, singlet oxygen, peroxynitrite anion, nitric oxide, and hypochlorous acid [28], and activate other antioxidative enzymes, such as glutathione peroxidase and superoxide dismutase [28]. Melatonin is able to prevent oxidative stress caused by mitochondrial fission [29, 30] and reverse mitochondrial damage by upregulating mitochondrial fusion [31]. Melatonin not only attenuates tight junction breakdown in the brain [32], but it also decreases retinal injury [33]. Furthermore, melatonin is able to reduce hepatic mitochondrial damage in both STZ- and obesity-induced diabetic rats [34, 35]. However, in diabetic patients, reports on intraocular melatonin are controversial. In diabetic patients, melatonin levels in the blood and retina are significantly decreased [36], which is correlated with increased insulin resistance [37]. On the contrary, increased melatonin is found in the aqueous humor of diabetic patients [38]. In the United States, melatonin can be self-administered and easily purchased without a doctor's prescription. About 0.7% of Americans use melatonin as a supplement, double that of 5 years ago [39]. Since there are contradicting reports on melatonin's action in retinal neurons [40–42], we aimed to clarify the efficacy of melatonin in preventing retinal dysfunction in early diabetes.

## 2. Materials and Methods

### 2.1. Cell Culture

Mammalian 661W cells were originally derived from a mouse retinal tumor and characterized as a cone-photoreceptor cell line, since they express cone-specific opsins, transducin, and arrestin [43, 44]. The 661W cells were obtained from Dr. Al-Ubaidi (University of Houston) and cultured in Dulbecco's modified Eagle's medium (DMEM) supplemented with 10% Fetal Bovine Serum (FBS), 1% Glutamax, and 1% antibiotics at 37°C and 5% CO_2_. Cultured 661W cells were treated with high glucose (HG, 30 mM) for different durations to examine any signal transduction changes. Some HG-treated cells were treated with melatonin (100 *μ*M) concurrently to determine the effect of melatonin on HG-induced changes.

### 2.2. Western Immunoblotting

Cell lysates were collected and prepared as described previously [45, 46]. Briefly, 661W cells were harvested and lysed in a Tris lysis buffer (in mM): 50 Tris, 1 EGTA, 150 NaCl, 1% Triton X-100, 1% *β*-mercaptoethanol, 50 NaF, and 1 Na_3_VO_4_, pH 7.5. Samples were separated on 10% sodium dodecyl sulfate-polyacrylamide gels by electrophoresis and transferred to nitrocellulose membranes. The primary antibodies used in this study were DRP1 (1 : 1000; Cell Signaling Technology, Danvers, MA, USA), MFN2 (1 : 1000 Abcam, Cambridge, MA, USA), MCU (1 : 1000 Abcam), and actin (loading control; 1 : 1000, Cell Signaling Technology). Blots were visualized using appropriate secondary antibodies (Cell Signaling Technology) at 1 : 1000 conjugated to horseradish peroxidase and an enhanced chemiluminescence (ECL) detection system (Pierce, Rockford, IL, USA). Band intensities were quantified by densitometry using Scion Image (NIH, Bethesda, MD, USA).

### 2.3. Calcium Imaging in Living Cells

The 661W cells were cultured on cover glass chambered slides (Nunc Lab-Tek; Thermo Fisher Scientific, Waltham, MA, USA) with the same medium described above. After treatment with HG or HG/melatonin for 24 hours, cells were directly loaded with 2 *μ*M Fluo-4 (Thermo Fisher Scientific) and 2 *μ*M rhodamine-2 (Rhod-2; Thermo Fisher Scientific) for 30 mins at 37°C for cytosolic and mitochondrial Ca^2+^ imaging [47, 48]. After washing, new culture medium was added, and then fluorescent images were taken under identical settings, including the light intensity, exposure time, and magnification. The average fluorescent intensity per pixel for each image was quantified without any modification using the luminosity channel of the histogram function of Photoshop 6.0 (Adobe Systems, San Jose, CA, USA). A total of 8 to 11 cell images from each group were analyzed from 3 different sets of experiments [45, 46].

### 2.4. Animals

Four-week-old wild-type (WT) male C57BL/6J mice were purchased from the Jackson Laboratory (Bar Harbor, ME, USA). All animal experiments were approved by the Institutional Animal Care and Use Committee of Texas A&M University. Mice were housed under temperature- and humidity-controlled conditions with 12 : 12 h light-dark cycles. All mice were given food and water *ad libitum*.

### 2.5. Diabetes Induction and Melatonin Treatment

At 5 weeks of age (body weight around 20 g), mice were randomly assigned to control or STZ-diabetic groups. The STZ-diabetic mice were given intraperitoneal (i.p.) injections of STZ (100 mg/kg body weight (b.w.)) for three consecutive days. STZ was first dissolved in 0.05 M citric buffer (pH 4.5; 10 mg/ml), and each mouse was injected at a dose of 200 *μ*l per 20 g b.w. The nondiabetic controls were given i.p. injections of citric buffer (same volume). The blood glucose levels were monitored once a week using a Clarity glucometer (Diagnostic Test Group, Boca Raton, FL, USA) during the daytime at Zeitgeber time (ZT) 10. One week post STZ injections, mice with a blood glucose level higher than 250 mg/dl were considered to be diabetic. At this time, half of the STZ-diabetic mice were given 10 mg/kg b.w. melatonin by oral gavage daily right before the room lights turned off for three months, while the other half were given H_2_O. Freshly prepared melatonin was mixed in H_2_O (2 mg/ml), and each mouse was given 100 *μ*l melatonin solution per 20 g b.w. Electroretinogram (ERG) recordings were used to record retinal light responses, and fluorescein angiography (FA) was used to monitor retinal vasculature changes for all mice monthly. STZ-diabetic mice were sacrificed after 3 months of melatonin treatments, and the eyes were fixed for further analyses.

### 2.6. *In Vivo* Electroretinogram (ERG)

The ERG retinal light responses were recorded as described previously [46]. Mice were dark-adapted for a minimum of 3 hours and anesthetized with an i.p. injection of Avertin (2% 2,2,2-tribromoethanol, 1.25% tert-amyl alcohol; Fisher Scientific, Pittsburgh, PA, USA) solution (12.5 mg/ml) at a dose of 500 *μ*l per 25 g of body weight. Pupils were dilated using a single drop of 1% tropicamide/2.5% phenylephrine mixture for 5 minutes. Mice were placed on a heating pad to maintain their body temperature at 37°C. The ground electrode was placed on the tail, and the reference electrode was placed under the skin in the cheek below the eye. A thin drop of Goniovisc (Hub Pharmaceuticals, Rancho Cucamonga, CA, USA) was applied to the cornea surface to keep it moist, and a threaded recording electrode conjugated to a minicontact lens (OcuScience, Henderson, NV, USA) was placed on top of the cornea. All preparatory procedures were done under a dim red light, and the light was turned off during the recording. A portable ERG device (OcuScience) was used to measure dark-adapted ERG recordings at light intensities of 0.1, 0.3, 1, 3, 10, and 25 candelas·second/meter^2^ (cd·s/m^2^). Responses to 4 light flashes were averaged at the lower light intensities (0.1, 0.3, 1.0, and 3.0 cd·s/m^2^), whereas only 1 light flash was applied for the higher light intensities (10 and 25 cd·s/m^2^). A 1-minute recovery period was programmed between different light intensities. The amplitudes and implicit times of the a- and b-waves were recorded and analyzed using the ERGView 4.4 software (OcuScience). Both eyes were included in the analyses.

### 2.7. Fluorescein Angiography (FA)

Mice were anesthetized with an i.p. injection of Avertin (12.5 mg/ml) at a dose of 500 *μ*l per 25 g of body weight. The pupils were dilated using a single drop of 1% tropicamide/2.5% phenylephrine mixture for 5 minutes. Immediately following pupil dilation, 10% sodium fluorescein (Akorn, Lake Forest, IL, USA) was i.p. injected at a dose of 50 *μ*l per 25 g of body weight. Images were taken using an iVivo Funduscope for small animals (OcuScience). The vascular parameters were further analyzed using Photoshop 6.0 (Adobe Systems) and AngioTool, an analytical software developed by the US National Institutes of Health/National Cancer Institute (Bethesda, MD, USA). Areas of 289 × 289 pixels in the peripheral retinal region (800 pixels from the optic nerve) were cropped using Photoshop and used to analyze the microvascular density using AngioTool. The primary retinal arteries and veins were not included in the analyses.

### 2.8. Immunofluorescent Staining

Mouse eyes were excised and prepared as previously described [46]. Briefly, the eyes were fixed with Zamboni fixative and processed for paraffin sectioning (4 *μ*m). Each glass slide contained single paraffin sections from the control (CON), STZ, and STZ plus melatonin (STZ+MEL) groups. After deparaffinization and antigen retrieval, sections were washed in phosphate-buffered saline (PBS), blocked with 10% goat serum for 2 hours at room temperature, and then incubated overnight with primary antibodies at 4°C. On the next day, sections were washed with PBS several times and incubated with fluorescent-conjugated secondary antibodies for 2 hours at room temperature and mounted with ProLong Gold antifade reagent containing 4′,6′-diamidino-2-phenylindole (DAPI; Invitrogen/Life Technologies, Grand Island, NY, USA). The primary antibodies used were DRP1, MFN2, and MCU. The secondary antibodies used were Alexa Fluor 488 goat anti-rabbit immunoglobulin G (IgG; 1 : 150 dilution; Molecular Probes/Life Technologies, Grand Island, NY, USA) and Cy5 goat anti-mouse IgG (1 : 150 dilution; Abcam). Images were obtained using a Zeiss Stallion digital imaging workstation equipped with a Zeiss Axiovert 200M microscope (Carl Zeiss AG, Oberkochen, Germany). Fluorescent images from each group were taken under identical parameters, including the same exposure time and magnification. Image analysis included the whole retina (from the photoreceptor outer segment to the ganglion cell layer), photoreceptor inner segments (“photoreceptors”), and the inner retina (from the inner nuclear layer to ganglion cell layer). The averaged fluorescence intensity per pixel for each image was quantified without any modification using the luminosity channel of the histogram function in Photoshop 6.0 (Adobe Systems), and the green or red fluorescence intensities were measured on a brightness scale from 0 to 255. A total of 3 to 5 retinal sections from each group were processed for immunostaining and image analyses. The fluorescent intensities of the control (CON) were arbitrarily set at 1 for each slide. *N* represents the number of mice from each group.

### 2.9. Statistical Analyses

All data are presented as mean ± standard error of the mean (SEM). Origin 8.6 software (OriginLab, Northampton, MA, USA) was used for statistical analyses. One-way analysis of variance (ANOVA) followed by Fisher's post hoc test was used for statistical analyses among all experimental groups. Both eyes from the same animal were used in the analyses, and “*n*” indicates the number of animals per group. Throughout, *p* < 0.05 was regarded as significant.

## 3. Results

### 3.1. High Glucose (HG) Induces Changes in Mitochondrial Fission/Fusion Dynamics and Intracellular Calcium Storage in Photoreceptor-Derived Cells

**Figure 1:**
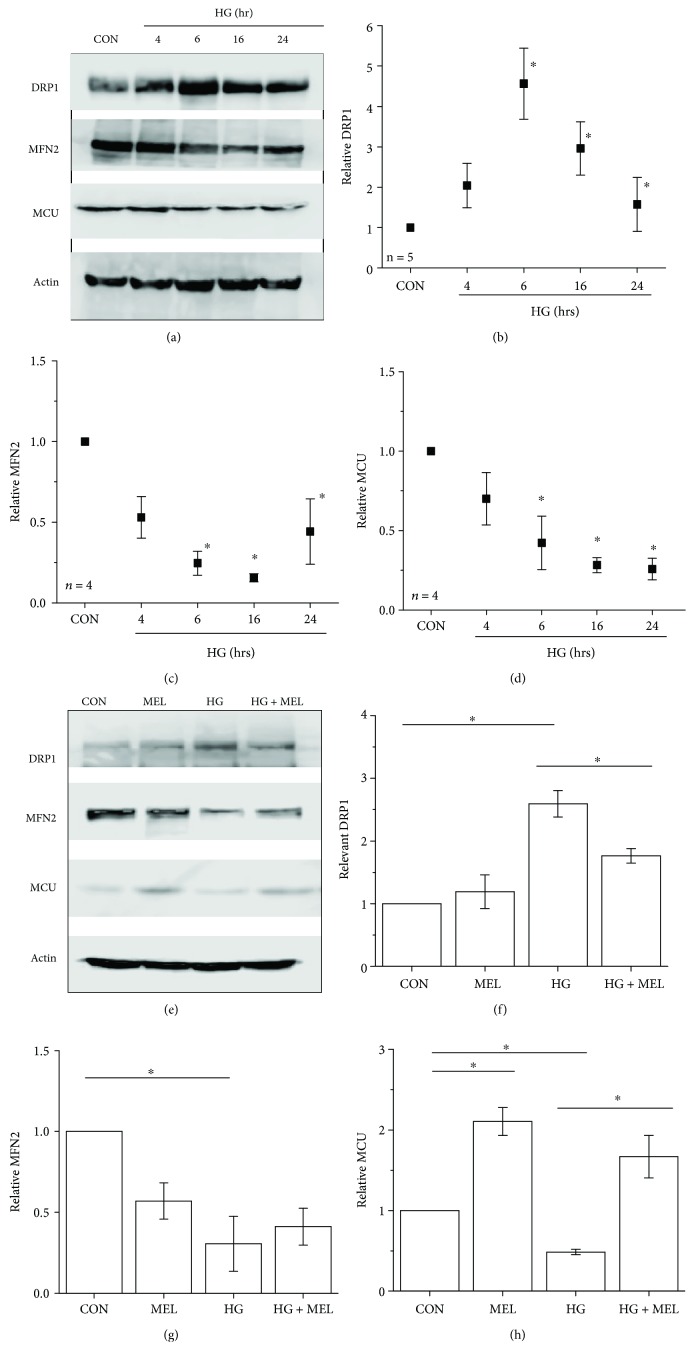
High glucose induces changes in mitochondrial fission/fusion dynamics in cultured 661W cells. Cultured 661W cells were acutely exposed to high-glucose conditions (HG; 30 mM) for 4, 6, 16, or 24 h (a-d) and collected for Western blotting of DRP1 (b), MFN2 (c), and MCU (d). The control (c) cells were treated with H_2_O. (e-g) Cultured 661W cells were treated with 0.01% ethanol (vehicle) as the control (CON), melatonin (MEL, 100 *μ*M; dissolved in 0.01% ethanol), HG (30 mM), or a combination of melatonin and HG (HG+MEL) for 24 h. Cells were then collected and processed for Western blotting analysis of DRP1 (f), MFN2 (g), and MCU (h). The experiments were repeated at least four times (*n* = 4-5). ^∗^*p* < 0.05.

Since photoreceptors are the largest cell population in the mouse retina and are the major source of intraocular oxidative stress in the diabetic retina [9], we used cultured 661W cells, a photoreceptor-derived murine cell line [44], to understand how hyperglycemia affects mitochondrial fission/fusion dynamics and whether melatonin is able to protect photoreceptors from hyperglycemia-induced damage in mitochondria. The cultured 661W cells were treated with high glucose (HG, additional 30 mM added into the culture medium) for 4, 6, 16, and 24 hours. Compared to the control (CON) treated with H_2_O (vehicle), treatments with HG upregulated DRP1 expression significantly from 6 to 24 hours but downregulated the expression of MFN2 from 6 to 24 hours in a time-dependent manner (Figure 1), indicating that HG induced mitochondrial fission but dampened the fusion process in cultured 661W cells. Treatments with HG also decreased the MCU expression in a time-dependent manner (Figure 1), indicating that HG might decrease the mitochondrial Ca^2+^ pool. Treatment with melatonin (100 *μ*M) concurrently with HG for 24 h was able to avert HG-caused increase of DRP1 and decrease of MCU (Figure 1), suggesting that melatonin might have protective actions against hyperglycemia-induced changes in mitochondrial fission-fusion dynamics and in decreased mitochondrial Ca^2+^ buffering capacity.

### 3.2. High Glucose- (HG-) Induced Decreases in the Mitochondrial Ca^2+^ Pool Are Alleviated by Melatonin Treatments

**Figure 2:**
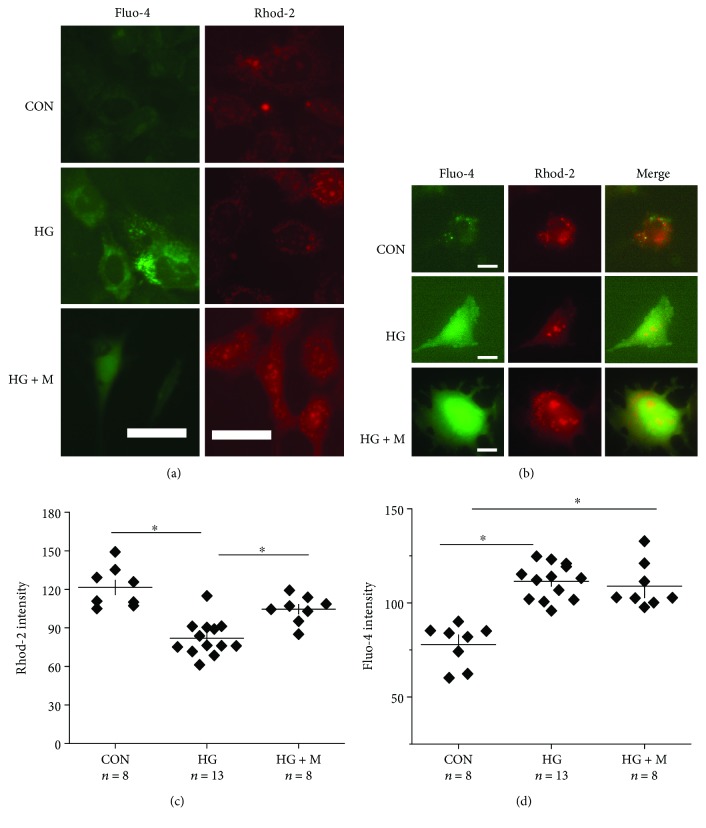
Melatonin treatments alleviate HG-induced decreases in mitochondrial Ca^2+^ buffering. Calcium-imaging of cultured 661W cells after 24 h of treatments with H_2_O (CON), HG, or HG+melatonin (HG+M). Cells were loaded with Fluo-4 and Rhod-2 for cytosolic and mitochondrial Ca^2+^ imaging, respectively. Scale bar = 20 *μ*m in (a) and 10 *μ*m in (b). The Rhod-2 (c) and Fluo-4 (d) fluorescent intensities indicating mitochondrial (c) and cytosolic (d) Ca^2+^ were quantified. The experiments were repeated at least three times. ^∗^*p* < 0.05.

Cultured retinal neurons [49] or retinal endothelial cells [50] treated with HG for a few days have an increase in cytosolic (intracellular) Ca^2+^, which can further lead to morphological changes in mitochondria [51] and cell apoptosis [50]. We postulated that HG-caused increase of cytosolic Ca^2+^ in part was due to the decrease of MCU (Figure 1), meaning mitochondria would have decreased capacity to store Ca^2+^. To verify this hypothesis, we used Fluo-4 [52] and Rhod-2 [53] to differentiate between cytosolic and mitochondrial Ca^2+^, respectively. Compared to the control (CON) treated with H_2_O, 661W cells treated with HG for 24 h had a higher Fluo-4 intensity with a decreased Rhod-2 intensity (Figure 2), reflecting that HG caused an increase of cytosolic Ca^2+^ but a decrease of mitochondrial Ca^2+^. The HG-induced decrease of the mitochondrial Ca^2+^ pool might partially contribute to the increased cytosolic Ca^2+^. We found that melatonin was able to prevent HG-caused decrease of MCU (Figure 1), indicating that melatonin might be able to alleviate HG-caused changes in the mitochondrial Ca^2+^ buffering ability. Melatonin treatment prevented HG-caused decreases in mitochondrial Ca^2+^ storage (Figure 2), but it did not completely dampen HG-induced increases in cytosolic Ca^2+^. These results (Figures 1 and 2) illustrate that HG-caused decrease of MCU dampened the mitochondrial capability to store Ca^2+^, and melatonin was able to prevent this impairment through an upregulation of MCU.

### 3.3. Melatonin Once-a-Day Oral Supplement Does Not Prevent STZ-Induced Diabetic Conditions Systemically

**Figure 3:**
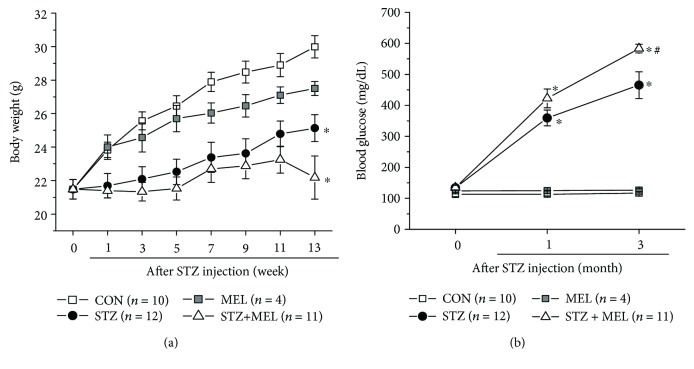
Daily treatments with melatonin did not improve STZ-induced diabetic conditions in body weights and systemic hyperglycemia. One week after STZ injections, some STZ-injected mice were given daily melatonin via oral gavage (STZ+MEL). (a) STZ-induced diabetic mice (STZ) with or without melatonin treatments gained weight more slowly starting 1 week after STZ injections compared to the control mice injected with citric buffer (CON). Three months after melatonin treatment, the average body weights of STZ+MEL mice or STZ mice were lower than those of the control mice (^∗^). There was no statistical difference between the CON and melatonin-treated (MEL) groups. (b) The STZ and STZ+MEL mice had significantly higher systemic blood glucose levels than the CON or MEL (^∗^). The blood glucose levels of STZ+MEL mice were higher than those of STZ mice (#) after 3 months post-STZ injection. ^∗^ denotes a statistical significance compared to the control mice (CON); # denotes a statistical significance compared to the STZ mice. ^∗^, #*p* < 0.05.

We used STZ injections to induce type 1 diabetes in this study. Mice were randomly assigned to four groups: the control (CON) injected with citric buffer, STZ-injected (STZ), melatonin-treated (MEL), and STZ-injected with daily melatonin treatments through oral gavage (STZ+MEL). Instead of providing melatonin in drinking water at all times or in daily intraperitoneal injections or by subdermal implants, which were used in published reports on the *in vivo* effects of melatonin, we administered 10 mg/kg b.w. of melatonin through oral gavage once daily to mimic the most commonly used intake route in humans. This dosage is equivalent to 0.7 mg/kg b.w. of humans [54], which is within the range of taking melatonin as a preventative treatment for cancer tumorigenesis [55] or management of insomnia [56, 57]. We administered melatonin once daily to mice right before the room light turned off (immediately before Zeitgeber time 12) to avoid circadian phase-shifting of the mice, as melatonin synthesis begins in the evening and continues throughout most of the nocturnal phase [58]. We monitored the body weights and blood glucose levels in mice before and after the STZ injections. Compared to the CON or MEL, STZ-induced diabetic mice had slower body weight gains (Figure 3(a)), and they developed diabetic hyperglycemia (above 250 mg/dl) within one month after the STZ injections (Figure 3(b)). Daily treatments with melatonin in STZ mice (STZ+MEL) did not improve the slow weight gain (Figure 3(a)). Chronic treatments with melatonin seemed to further worsen the hyperglycemic condition in STZ-diabetic mice (Figure 3(b)). Hence, daily melatonin treatment through oral gavage was not effective in controlling systemic glycemia in STZ-induced diabetic mice.

### 3.4. Dark-Adapted Retinal Light Responses Are Decreased in Diabetic Mice Three Months after STZ Injections

Distorted color vision and delayed retinal light responses are among the first clinical signs of retinal dysfunction in early stage diabetic patients without DR [59, 60]. We previously reported that the retinal light responses are delayed in obese mice that were given a high-fat diet for only one month, even though at this point these mice still have normal blood glucose levels [46]. This result verifies that under prediabetic conditions, the physiology of the neural retina might have been compromised. Melatonin treatments through either intraperitoneal injections [61] or subcutaneous implantation of melatonin pellets [33] in STZ-diabetic rats improve STZ-induced reduction of retinal light responses. We next examined at what point does the STZ-induced hyperglycemic condition cause retinal dysfunction and whether melatonin treatments through daily oral gavage might have the same beneficial effects as other routes by using ERG recordings to measure retinal light responses monthly.

**Figure 4:**
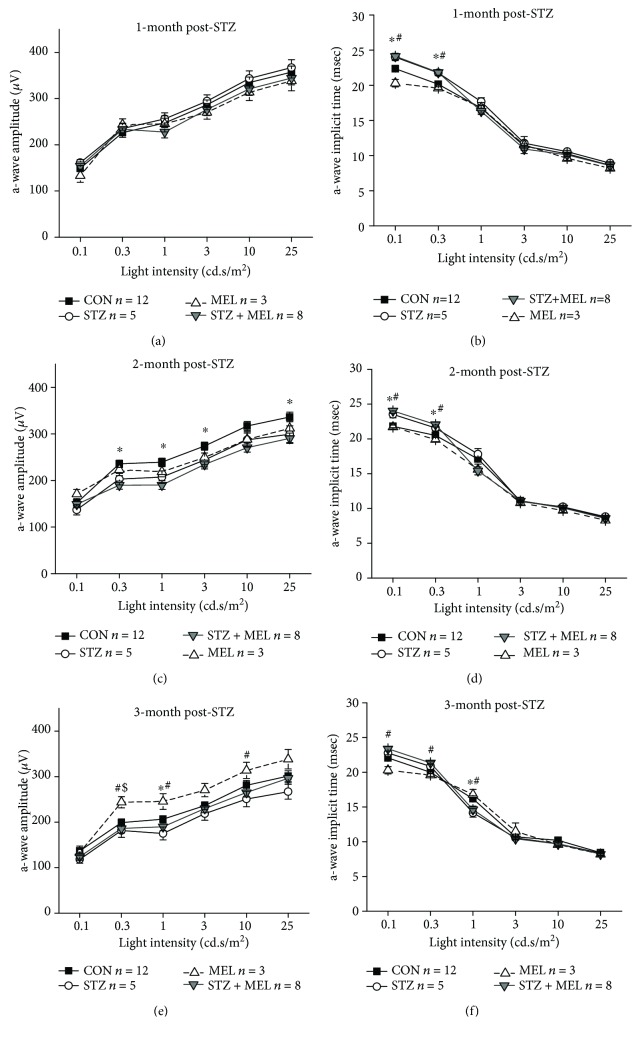
Dark-adapted ERG a-wave amplitudes and implicit times recorded at one, two, and three months after STZ injections. The dark-adapted ERG a-wave amplitudes (a, c, e) and implicit times (b, d, f) in the control (CON), STZ-injected (STZ), melatonin-treated (MEL), and STZ-injected and melatonin-treated (STZ+MEL) mice at 1 month (a, b), 2 months (c, d), and 3 months (e, f) after the STZ injections. ^∗^ denotes a statistical significance between CON and STZ; # denotes a statistical significance between MEL and STZ+MEL; $ denotes a statistical significance between CON and MEL; ^∗^*p* < 0.05.

**Figure 5:**
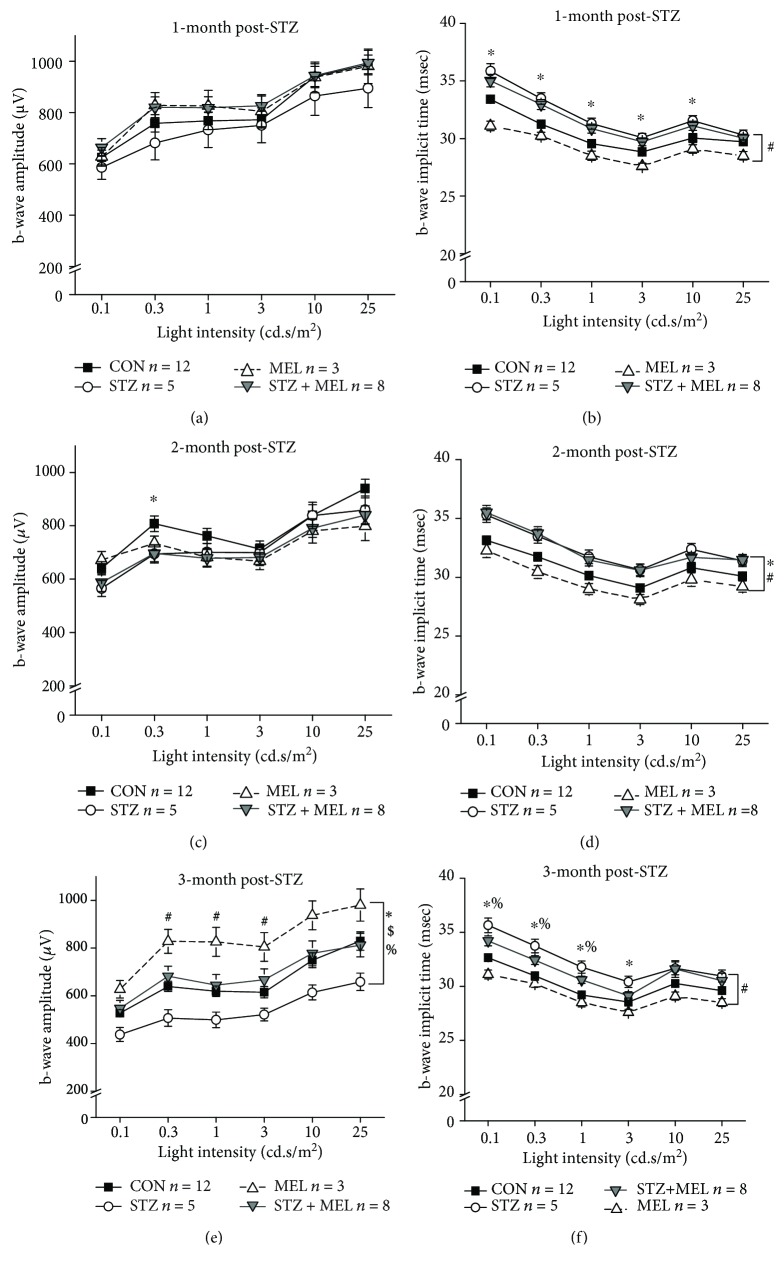
Dark-adapted ERG b-wave amplitudes and implicit times recorded at one, two, and three months after STZ injections. The dark-adapted ERG b-wave amplitudes (a, c, e) and implicit times (b, d, f) in the control (CON), STZ-injected (STZ), melatonin-treated (MEL), and STZ-injected and melatonin-treated (STZ+MEL) mice at 1 month (a, b), 2 months (c, d), and 3 months (e, f) after the STZ injections. ^∗^ denotes a statistical significance between CON and STZ; # denotes a statistical significance between MEL and STZ+MEL; $ denotes a statistical significance between CON and MEL; % denotes a statistical significance between STZ and STZ+MEL; ^∗^*p* < 0.05.

Mice were first dark adapted for at least 3 h prior to the ERG recordings with stimulations of various light intensities at 0.1, 0.3, 1, 3, 10, and 25 cd·s/m^2^ (Figures 4 and 5). The ERG a-wave reflects the light responses from the photoreceptors, while the b-waves reflect the inner retinal light responses [62]. The ERG implicit times reflect how fast the neural retina responds to light flashes [63]. One month after STZ injections, STZ-injected mice without melatonin treatments (STZ) had delayed retinal light responses, as their implicit times were longer compared to the non-STZ mice (Figures 4 and 5). This delayed light response in the STZ mice continued at 2- and 3-month post STZ injections, along with significantly lower light responses as reflected by decreased a- and b-wave ERG amplitudes (Figures 4(c)–4(f) and 5(c)–5(f)), indicating that chronic hyperglycemic conditions negatively impact retinal light responses. Treatments with melatonin for 3 months (MEL) seemed to increase the retinal light responses in nondiabetic mice, and melatonin treatments (STZ+MEL) appeared to prevent STZ-induced dampening of inner retinal light responses (b-wave) by this time, since the ERG b-wave amplitudes of the STZ+MEL group were similar to that of the control (CON; Figure 5(e); % denotes a statistical significance between STZ and STZ+MEL). Hence, through once daily oral gavage treatments for 3 months, melatonin appears to have protective effects on retinal light responses against STZ-induced diabetes.

### 3.5. Melatonin Appears to Prevent the Development of STZ-Induced Microvascular Complications

**Figure 6:**
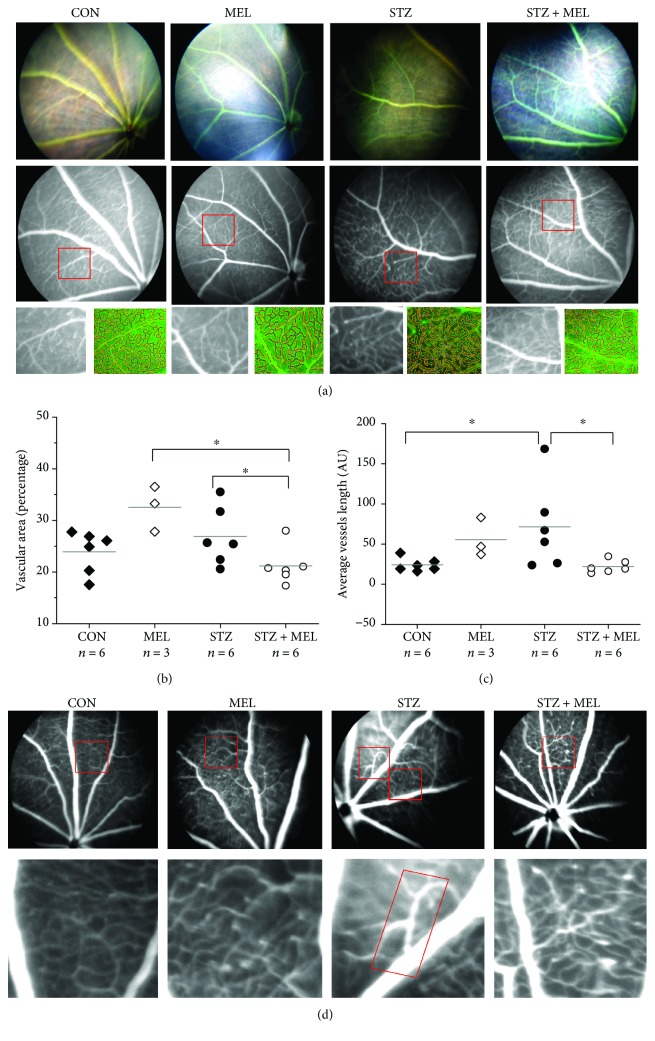
Melatonin treatments appear to prevent the development of microvascular complications. (a) Fluorescein angiography was used to visualize the intraocular vasculature in mice. AngioTool was used to determine the (b) percentage of vascular area to the retinal area and (c) the average vessel length. “AU” is the arbitrary unit used in the AngioTool software. The vascular area (percentage) and the average vessel length in STZ+MEL mice are significantly less than those in the STZ mice (^∗^). (d) Venous beading (rectangle) was observed in STZ mice. ^∗^*p* < 0.05.

We previously reported that high-fat diet-induced diabetic mice have increased microvascular complications including increased vascular permeability (shown as increased vascular areas) and acellular microvasculature in the peripheral retina at 6-7 months after the high-fat diet regimen [46, 64, 65]. There is increased vascular permeability in STZ-induced diabetic mice [66]. Even though melatonin appeared to have a temporary and mild effect on retinal light responses of diabetic mice (STZ+MEL; Figures 4 and 5), we next examined whether melatonin had any protective effect on STZ-induced microvascular changes. We employed fluorescein angiography (FA), a tool that reveals changes in the ocular vasculature and can indicate vascular permeability in the eye [67], with AngioTool (NIH) to visualize and quantify the ocular vessels (Figure 6). We previously did not find any major vascular changes in the central retina in obesity-induced diabetic animals [46, 64, 65]. As such, we focused on vascular changes in the peripheral retina. These STZ mice (3 months after STZ injections) had an increase in vascular area and average vessel length (Figure 6), which echoes a previous finding that there is an increase in ocular vascular permeability at 3 months after STZ injections [66]. Daily treatments with melatonin had a dampening effect in STZ-induced increases in vascular area and average vessel length (Figure 6). As daily oral gavage of melatonin (MEL) did not affect body weight, blood glucose levels (Figure 3), or retinal light responses (Figures 4 and 5), unexpectedly, melatonin alone (MEL) appears to increase the vascular area (Figure 6), even though treatments with melatonin in STZ mice (STZ+MEL) decreased the vascular area and average vessel length. We also observed “venous beading” in 3 of the 6 STZ mice at 3 months post STZ injections (Figure 6(d), STZ, red rectangle). Venous beading is a microvascular abnormality often observed in the eyes of patients with nonproliferative DR [68]. Venous beading was not observed in the control (CON), MEL, or STZ+MEL mice, indicating that melatonin treatments might avert venous beading in STZ-diabetic mice. Thus, treatments with melatonin might be able to prevent the STZ-induced microvascular complications in the retina.

### 3.6. Melatonin Administration Alleviates the STZ-Disturbed Mitochondrial Fission-and-Fusion Dynamics and Calcium Buffering in the Diabetic Retina

**Figure 7:**
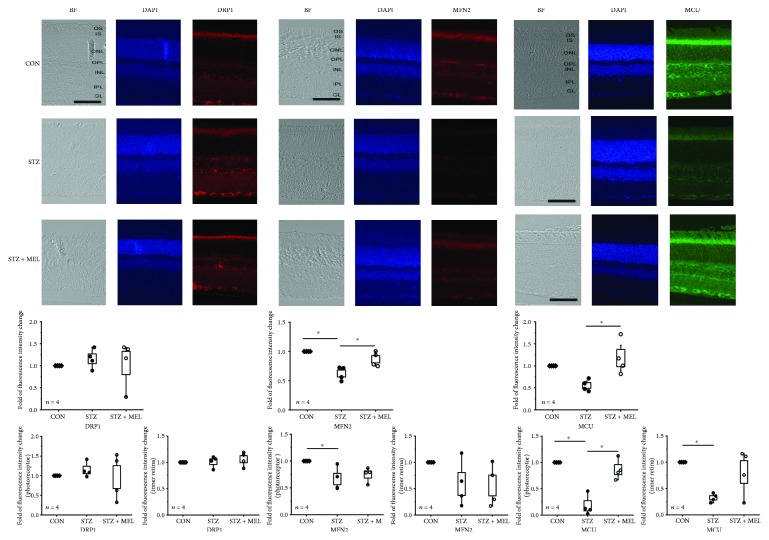
Daily melatonin treatments prevent STZ-induced changes in mitochondrial fusion and Ca^2+^ uniporter proteins. The immunofluorescent images of DRP1, MFN2, and MCU in the neural retinas from three groups are shown. The analyzed data of relative changes in fluorescent intensities of the whole retina (second row), the photoreceptors (IS only; third row), and the inner retina (from INL to GL; third row) are shown. The fluorescent intensities of the control (CON) were arbitrarily set at 1 for each slide. Each datum point is the average of relative fluorescent intensities (multiple images) from a single mouse. There is no apparent change of DRP1 in the STZ mouse retina (STZ) compared to the control (CON). Melatonin does not have an impact on DRP1 in STZ mouse retinas (STZ+MEL). The STZ mouse retina (STZ) has an apparent decrease in MFN2 compared to both CON and melatonin-treated (STZ+MEL) groups in the photoreceptors. The STZ mouse retina (STZ) also has a significantly decreased MCU expression compared to the melatonin (STZ+MEL) group in the photoreceptors and inner retina. BF = Bright field. DAPI stained nuclei. Scale bar = 50 *μ*m. OS: photoreceptor outer segments; IS: photoreceptor inner segments; ONL: outer nuclear layer; OPL: outer plexiform layer; INL: inner nuclear layer; IPL: inner plexiform layer; GL: ganglion cell layer. ^∗^*p* < 0.05.

As mentioned previously, mitochondria undergo fission processes when cells are under nutrient overload in cultures [4, 5]. We showed that when 661W cells are cultured in high-glucose conditions (Figure 1), there was an increase of DRP1 and decrease of MFN2 indicating an increase of mitochondrial fission but a decrease in fusion. Since melatonin might have a protective effect in diabetic retinas, we examined whether the mitochondrial fission-and-fusion dynamics in the retina might be altered under chronic hyperglycemic conditions with a focus on the mitochondrial changes in the retina from STZ mice with or without melatonin treatments. Interestingly, we found that there was no significant change in the mitochondrial fission process in the retina measured by the immunostaining of DRP1 in the STZ-diabetic mouse retina (STZ) compared to the control (CON), and treatment with melatonin in STZ mice (STZ+MEL) did not have any impact on the retinal DRP1 expression (Figure 7). However, the mitochondrial fusion process measured by the immunostaining of MFN2 is significantly decreased in the STZ-diabetic mouse retina, and the decrease was more profound in the photoreceptors than in the inner retina, but the MFN2 level in the STZ mouse retina treated with melatonin (STZ+MEL) was similar to that of the control (CON), indicating that melatonin might prevent STZ-induced decrease of mitochondrial fusion in the retina.

In diabetic cardiomyocytes, mitochondrial Ca^2+^ is decreased by 40%, and the buffering capacity of mitochondria is altered [69]. Since STZ mouse retinas had altered levels of the mitochondrial fusion protein MFN2, we next examined whether the expression of MCU was also altered, since MCU is responsible for storing intracellular Ca^2+^ in the mitochondria [15, 16]. Similar to the cultured 661W cells that were under hyperglycemic conditions which had decreased MCU and mitochondrial Ca^2+^ pools (Figures 1 and 2), we found that the protein expression of MCU was decreased in the STZ mouse retinas (photoreceptors as well as the inner retina), but treatments with melatonin in these mice (STZ+MEL) were able to prevent the STZ-induced loss of MCU in photoreceptors as well as the inner retina (Figure 7), implying that melatonin treatments could protect or recover mitochondrial Ca^2+^ buffering ability in the diabetic retina.

## 4. Discussion

Maintaining proper mitochondrial fission/fusion dynamics is critical for keeping cells healthy. When there is a nutrient overload, such as in hyperglycemic or diabetic conditions, it causes an imbalance in mitochondrial dynamics and leads to oxidative stress. Melatonin is an antioxidant and is also known to inhibit proangiogenic factors, relieve oxidative stress and inflammation [61, 70, 71], and rescue retinal damage in diabetic animals [33, 61, 72]. Melatonin is able to prevent oxidative stress caused by chemically induced mitochondrial fission [29, 30] and reverse ROS-induced mitochondrial damage by upregulating mitochondrial fusion [31], but we report here for the first time that melatonin is able to prevent hyperglycemia-caused decrease of mitochondrial fusion and Ca^2+^ pool both *in vitro* and *in vivo*. Even though there are multiple protein complexes involved in the mitochondrial fission/fusion dynamics and Ca^2+^ buffering, we selected key players that are essential for these processes (DRP1, MFN2, and MCU) to investigate the effects of melatonin in hyperglycemia-caused changes in mitochondrial function, as well as the protective effect of melatonin in STZ-induced diabetic retinas. As overexpression of MCU can restore the damage caused by hyperglycemia-associated oxidative stress in cardiomyocytes [73], our data showed that melatonin prevented hyperglycemia-caused decreases of MCU in cultured 661W cells and STZ-diabetic retinas, which implies that melatonin treatments could decrease the damage caused by diabetes-induced oxidative stress in part through protecting mitochondria Ca^2+^ buffering ability in the retina.

Interestingly, we found that daily melatonin treatment through oral gavage was not effective in controlling systemic glycemia in STZ-induced diabetic mice (Figure 3), which echoes previously published data using subcutaneous implants of melatonin pellets to treat STZ-induced diabetic rats [33]. The melatonin-insulin antagonism might explain the ineffectiveness of chronic melatonin treatments on controlling systemic glucose levels. Healthy rats given melatonin through drinking water for 3 months have decreased serum insulin but increased corticosterone in addition to altered metabolic profiles [74]. If exogenous melatonin further decreases systemic insulin in STZ-diabetic mice that already secrete extremely low insulin [75], it would lead to a failure to control systemic glycemia.

Diabetic retinopathy is a dual disorder with microvascular complications and retinal degeneration [19]. Monthly ERG recordings showed that retinal light responses changed in mice at 1-month post-STZ injections as their implicit times increased, which shows that the health of the neural retina might have been compromised before any detectable vascular complications. However, one potential concern is the effect of STZ itself. Streptozotocin is a well-known chemical that induces type 1 diabetes in animals through targeting the islet cells, but it is also toxic to the neural retina [76]. Within a month after STZ injections, there is transient cell apoptosis and upregulated glial activation in the neural retina, but these abnormalities quickly return to normal in the subsequent 2-4 months [77]. Further retinal degeneration and acellular capillaries are observed at least 6 months after the STZ injections [77]. This indicates that STZ might have a transient toxic effect on the neural retina within the first few weeks of injections, which is also reflected in our overall ERG a- and b-wave amplitudes. However, the decreased b-wave amplitudes and increased implicit times after 3 months of STZ injections might indicate that hyperglycemic conditions worsen the retinal light responses.

We found that melatonin treatments had a protective effect on the neural retina particularly after 3 months of treatments. Previous studies using daily intraperitoneal (i.p.) injections of melatonin (10 mg/kg) [61] or melatonin pellet implants (20 mg) [33] to treat STZ-induced diabetic rats demonstrated that these treatments for 3 months clearly alleviate the STZ-induced decreases in ERG a- and b-wave amplitudes back to the normal levels of nondiabetic rats, which are similar to our results. However, treatment with melatonin alone for 3 months significantly enhanced ERG a- and b-wave amplitudes in mice (Figures 4 and 5) but not in rats [33]. There are two possible explanations for the discrepancy between our results and previous reports on the effects of melatonin on retinal light responses. First, the retinal sensitivity to melatonin treatments is species-dependent. As such, rats might be more responsive to melatonin than the mouse strain (C57BL/6J) that was used in our study. The C57BL/6J is the most used mouse strain to study retinal function since it does not carry genes that cause retinal degeneration. However, C57BL/6J is melatonin-deficient, since this mouse strain lacks serotonin-*N*-acetyltransferase and hydroxyindole-*O*-methyl-transferase, two enzymes needed for the synthesis of melatonin from serotonin [78], and it is not clear whether the retinal expression of melatonin receptors in C57BL6J is similar to that of other melatonin-proficient animals [79]. It is possible that the retina of melatonin-proficient animals (such as rats) might respond to exogenous melatonin more effectively compared to that of melatonin-deficient animals. Second, the routes of treatments and the dosages between our current study (oral) and previous reports (i.p. [61] or subcutaneous implantation [33]) are different. We strived to mimic the most common route of human intakes of melatonin (orally, once a day) at a comparable dosage. Thus, it is possible that our overall melatonin dosage absorbed by the animals was not as much compared to those in previous reports.

We also found that melatonin had a protective effect on the retinal microvasculature. We used FA to chronologically monitor the vascular changes, and increases in average vessel length were observed in mice at 3 months post STZ injections. Even though we did not specifically perform vascular permeability assays, changes in FA could indicate vascular permeability in the eye [67]. Venous beading was observed in 50% of the STZ-diabetic mice, but STZ mice treated with melatonin (STZ+MEL) did not have venous beading despite their diabetic status. Melatonin treatments also dampened STZ-induced increases in average vessel length, which indicates that melatonin treatments might prevent vascular permeability in diabetic animals. This indicates that at our dosage via oral gavage, melatonin might have direct effects on the microvasculature, in which the molecular action of melatonin on the microvasculature requires further investigation.

The ATP level is critical in regulating mitochondrial dynamics [45]. In cultured retinal cells, treatment with HG elevates the extracellular ATP, where the release of ATP is involved in the inflammation process [80]. We found that treatments with HG elevated DRP1 and dampened MFN2 in cultured 661W photoreceptors, and the level of MFN2 was also decreased in the STZ-diabetic retina. Mitochondrial fission and fusion play a crucial role in regulating energy expenditure and oxidative metabolism. Tissue-specific ablation of MFN2 in the liver impairs insulin signaling and increases hepatic gluconeogenesis and endoplasmic reticulum (ER) stress [81]. We found that melatonin treatments were able to prevent the STZ-induced decrease of MFN2 in the retina and the HG-caused elevation of DRP1 in cultured 661W cells. These data imply that melatonin is able to avert diabetes-induced decreases in mitochondrial fusion and HG-caused increases of mitochondrial fission. One possible mechanism is that melatonin blocks the translocation of DRP1 into mitochondria to prevent their fission [82]. Hence, melatonin could alleviate hyperglycemia-induced changes in mitochondrial dynamics.

In diabetic pancreases [83] and hearts [84], mitochondria function is disturbed due to the downregulation of MCU. Our data confirmed that the expression of MCU was decreased in HG-treated 661W cells and STZ-diabetic retinas. Further, hyperglycemia decreased the mitochondrial calcium buffering capability in these cells. Treatment with melatonin prevented HG-caused decreases in mitochondrial Ca^2+^ in part through increasing the MCU expression. The decrease of retinal mitochondrial Ca^2+^ buffering ability affects the mitochondrial dynamics [85] and potentially worsens the progression of DR [86]. Thus, our data provide evidence that melatonin is able to recover hyperglycemia-induced decrease of mitochondrial Ca^2+^ pools by increasing the expression of MCU. However, melatonin treatments did not dampen the HG-induced increase of cytosolic Ca^2+^. Since the overall cytosolic Ca^2+^ depends on the calcium influx through various calcium channels in the plasma membrane, the calcium ATPase in the plasma membrane to extrude intracellular Ca^2+^, the mitochondrial Ca^2+^ pool, and the ER Ca^2+^ storage, we postulate that melatonin is not able to recover all of the components regulating intracellular calcium homeostasis.

Whether the effect of melatonin on mitochondria is through its antioxidant property or through its receptors remains to be determined, since the interaction between melatonin and its receptors depends on the exposure dosage, duration, and cell types. Treatment with melatonin (100-1000 nM) prevents H_2_O_2_-induced photoreceptor death in part through melatonin receptors [87]. When an ovary cell line is exposed to a lower concentration of melatonin (400 pM) for 8 hours, an increase in type 1 melatonin (MT1) receptor binding sites occurs [88]. However, treating the same ovary cells at a higher concentration of melatonin (1 *μ*M) for 5 hours desensitizes the MT1 receptors and inhibits phosphoinositide hydrolysis and the subsequent signal transduction cascade [89]. Intraperitoneal injections with 10 mg/kg melatonin cause melatonin to accumulate in the mitochondria much more than in the cytosol, which is not mediated by the MT1 or MT2 receptors on the plasma membrane [90]. This result implies that melatonin at higher concentrations could act directly in the mitochondria, in which we showed that melatonin alone affected MCU and MFN2. Melatonin might also directly act inside the cells as a scavenger of reactive oxygen species or to rescue mitochondria when cells are under oxidative stress. However, MT1 receptors appear to be expressed in the mitochondria isolated from brain lysates [91], and mitochondria also have the ability to synthesize melatonin in brain neurons [92]. Thus, whether the action of melatonin on mitochondria in photoreceptors is mediated by the mitochondrial MT1 receptors remains to be determined.

Taken together, we showed that melatonin is able to avert hyperglycemia-induced changes in mitochondrial dynamics and Ca^2+^ storage in cultured 661W cells. We demonstrated that neural retinal dysfunction might precede any detectable microvascular complications in type 1 diabetes. While the efficacy of melatonin in treating human diabetes still requires more in-depth studies, using an equivalent daily dosage of melatonin (0.7 mg/kg b.w.) taken orally might have a protective effect against diabetes-induced retinal dysfunction and prevent diabetic microvascular complications.

## Data Availability

The data (numbers, images, and Western blots) used to support the findings of this study are included within the article. The raw/quantified data used to support the findings of this study are available from the corresponding author upon request.
